# Complete mitochondrial genome of *Atergatis integerrimus* (Decapoda, Xanthidae) from the Philippines

**DOI:** 10.1080/23802359.2018.1437833

**Published:** 2018-02-10

**Authors:** Mustafa Zafer Karagozlu, Michelle M. Barbon, Thinh Do Dinh, Cesar G. Demayo, Chang-Bae Kim

**Affiliations:** a Department of Biotechnology, Sangmyung University, Seoul, Korea;; b Department of Biological Sciences, College of Science and Mathematics, Mindanao State University, Iligan Institute of Technology, Iligan City, Philippines

**Keywords:** Arthropoda, Decapoda, Xanthidae, complete mitochondrial genome, *Atergatis integerrimus*

## Abstract

In this study, a complete mitochondrial genome from the red egg crab, *Atergatis integerrimus* was sequenced and analyzed. The mitochondrial genome length is 16,333 bp and is composed of 13 protein-coding, two ribosomal RNA and 22 tRNA genes. The structure and gene orientation of the mitochondrial genome was found to be identical with the other brachyurans. The position of *A. intergerrimus* in the superfamily Xanthoidea was determined based on the mitochondrial protein-coding genes. The result of the phylogenetic relationship study showed that *A. integerrimus* was closest to *Leptodius sanguineus.*

The primary tool for identification of brachyuran is usually based on classical taxonomy mostly on morphological attributes. The red egg crab, *Atergatis integerrimus* is sometimes mistaken for stone crabs *Menippe rhumpii* because of morphological similarity (Vartak et al. 2015). It was, therefore, the objective of this study to provide a complete mitogenome of toxic crab *Atergatis integerrimus* from the Philippines and determine its position in the phylogenetic tree of the family Xanthidae by using the amino acid sequences of the mitochondrial protein-coding genes.

The specimen of *A. integerrimus* was collected from Agusan del Norte in the Philippines (8°59′25.98″N 125°19′17.97″E) on April 2017 and identified based on its DNA barcode. The specimen was stored in Department of Biotechnology, Sangmyung University, Korea (SM00250). NGS sequencing sequencing was subjected to the gDNA (Miseq, Illumina, San Diego, CA) and the paired end reads of mitogenome sequences were assembled and annotated using MITObim (Hahn et al. 2013), MITOS (Bernt et al. 2013) and ARWEN (Laslett and Canbäck 2008). The phylogenetic tree of Xanthidae was reconstructed based on concatenated amino acid sequences of 13 mitochondrial protein coding genes using the software MEGA 7.0 (Kumar et al. 2016).

The complete mitogenome genome size of *A. integerrimus* is 16,333 bp with 32.9% A, 20.9% C, 10.5% G and 35.7% T nucleotide distrubition (GenBank accession numer: MG792342). It consist 37 genes which are 13 protein-coding genes, two ribosomal RNA genes and 22 tRNA. The orientations of all these genes were typical of all brachyuran species (Miller et al. 2004). It was also shown that the mitogenome have 14 overlapping regions consisting of 1–25 bp in length where the longest overlapping region was found located between *nad1* and *tRNA^Leu^*. Results also revealed the mitogenome of *A. integerrimus* has 16 intergenic sequences varying from 1 to 633 bp in length where the largest intergenic sequence was found to be located between 12S rRNA and *tRNA^Val^*.

The reconstruction of the phylogenetic tree of superfamily Xanthoidea to determine the position of *A. integerrimus* show it is closest to the species *Leptodius sanguineus* (KT896744), the only xanthid crab recorded (Figure 1). Also Bythograeoidea is the closest superfamily to Xanthoidea which were declared by previous study as well (Tsang et al. 2014). Based on previous studies combining the use of nuclear (28S and H3) and mitochondrial (12S rRNA, 16S rRNA and COI) genes showed that while the superfamily Xanthoidea is monophyletic, the family Xanthidae is observed to be paraphyletic (Thoma et al. 2013). It is, however, important to note that the complete mitogenome records are not enough to confirm this evolutionary hypothesis. The mitogenome records of Xanthid crabs are still limited to confirm their phylogenetic relationship in this superfamily. Additional complete mitochondrial genome data from a majority if not all of the species in this genus should be made available to provide a complete picture of the phylogenetic relationships of the different species. The present study, however, provides additional data in understanding the molecular genetic basis of Xanthidae phylogeny.

**Figure 1.**
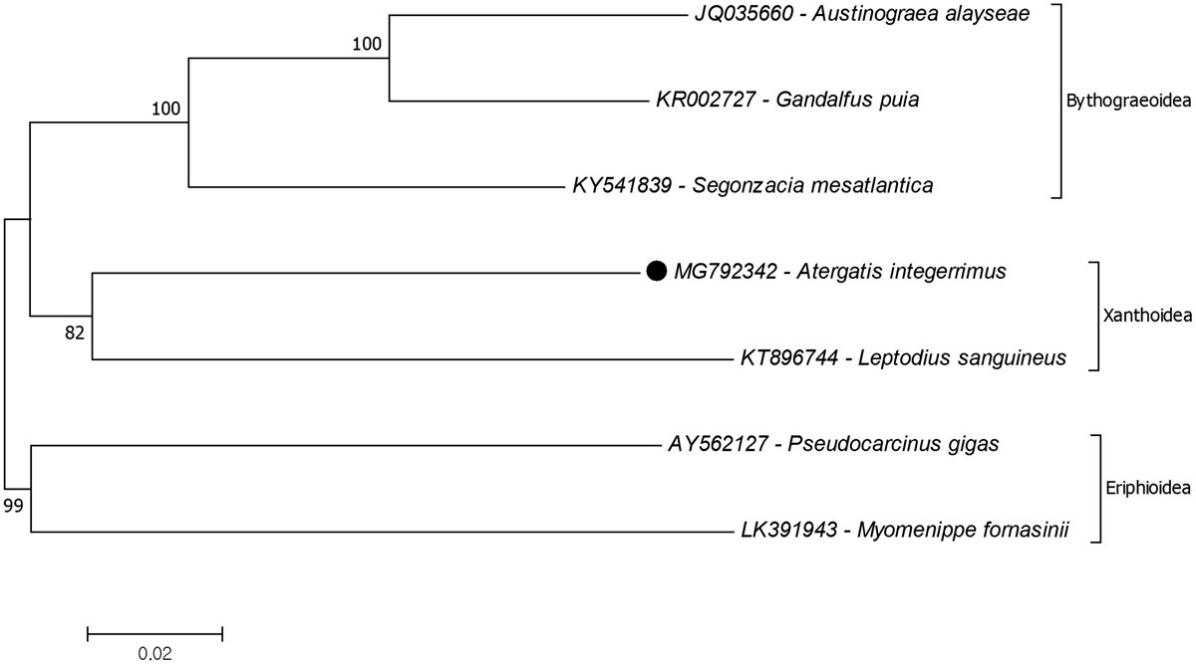
Phylogenetic relationships of that Atergatis integerrimus (black dot) in the superfamily Xanthoidea due to amino acid sequences of mitochondrial protein coding genes. The mitochondrial genome data retrieved from GenBank.
